# Decay of *TRPV3* as the genomic trace of epidermal structure changes in the land‐to‐sea transition of mammals

**DOI:** 10.1002/ece3.8731

**Published:** 2022-03-18

**Authors:** Tianzhen Wu, Luoying Deme, Zhenhua Zhang, Xin Huang, Shixia Xu, Guang Yang

**Affiliations:** ^1^ 12534 College of Life Sciences Jiangsu Key Laboratory for Biodiversity and Biotechnology Nanjing Normal University Nanjing China; ^2^ Southern Marine Science and Engineering Guangdong Laboratory Guangzhou China

**Keywords:** inactivating mutations, land‐to‐sea transition, epidermal structure, mammals, relaxed selective pressure, *TRPV3*

## Abstract

The epidermis plays an indispensable barrier function in animals. Some species have evolved unique epidermal structures to adapt to different environments. Aquatic and semi‐aquatic mammals (cetaceans, manatees, and hippopotamus) are good models to study the evolution of epidermal structures because of their exceptionally thickened stratum spinosum, the lack of stratum granulosum, and the parakeratotic stratum corneum. This study aimed to analyze an upstream regulatory gene transient receptor potential cation channel, subfamily V, member 3 (*TRPV3*) of epidermal differentiation so as to explore the association between *TRPV3* evolution and epidermal changes in mammals. Inactivating mutations were detected in almost all the aquatic cetaceans and several terrestrial mammals. Relaxed selective pressure was examined in the cetacean lineages with inactivated *TRPV3*, which might contribute to its exceptionally thickened stratum spinosum as the significant thickening of stratum spinosum in *TRPV3* knock‐out mouse. However, functional *TRPV3* may exist in several terrestrial mammals due to their strong purifying selection, although they have “inactivating mutations.” Further, for intact sequences, relaxed selective constraints on the *TRPV3* gene were also detected in aquatic cetaceans, manatees, and semi‐aquatic hippopotamus. However, they had intact *TRPV3*, suggesting that the accumulation of inactivating mutations might have lagged behind the relaxed selective pressure. The results of this study revealed the decay of *TRPV3* being the genomic trace of epidermal development in aquatic and semi‐aquatic mammals. They provided insights into convergently evolutionary changes of epidermal structures during the transition from the terrestrial to the aquatic environment.

## Graphical abstract

Inactivating mutations were detected in TRPV3 of almost all the aquatic cetaceans and several terrestrial mammals. The loss of TRPV3 may contribute to its exceptionally thickened stratum spinosum in the cetacean lineages. Relaxed selective constraints on TRPV3 gene were convergently detected in three clades (cetaceans, manatees, and hippopotamus) with similar epidermis structure, which may promote adaptation to the aquatic environment.

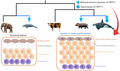

## 1 INTRODUCTION

The epidermis acts as a stable environmental barrier. In most mammals, the epidermis includes stratum basale, stratum spinosum, stratum granulosum, and stratum corneum (Simpson et al., 2011). It performs multiple protective functions in organisms, such as preventing water loss, excluding toxins, resisting mechanical stresses, and participating in immune responses. Multiple mammalian lineages have independently evolved specialized epidermal structures to adapt to different habitats. Terrestrial mammals generally evolved a hair coat to protect the skin from mechanical insults and facilitate homeothermy. Hence, the epidermis of these animals is fairly thin. As completely aquatic mammals, cetaceans have exceptionally thickened stratum spinosum that results in significantly increased thickness of the whole epidermis. However, they lack stratum granulosum and develop distinctive parakeratotic stratum corneum with flattened cells retaining pyknotic nuclei (Japha, 1905; Spearman, 1972). Similarly, a granular layer was not found in the fully aquatic manatee (Jarrett et al., 1965). The parakeratotic stratum corneum is rich in phospholipid in cetaceans and manatees, which probably helps waterproof the epidermis (Cane & Spearman, 1969; Spearman, 1970). Also, the thickening of the stratum spinosum enhances their immunity and wound‐healing ability (Simpson et al., 2011). For these aquatic mammals, the keratin layer is perhaps not needed for protection from the wound. In contrast, for the semi‐aquatic hippopotamus (*Hippopotamus amphibius*), the stratum granulosum is ill‐defined. Few cells contain the typical granules and are seldom densely packed (Luck & Wright, 1964), similar to that in cetaceans. Another semi‐aquatic marine carnivorans, pinnipeds, have dense coats and do not show parakeratosis (Sokolov, 1960). The epidermis structure of pinnipeds does not differ from that of land mammals, which is perhaps associated with the fact that they have to come ashore to mate, give birth, molt, or escape from predators. However, the molecular mechanisms underlying the evolutionary changes in the epidermal structure in different mammals have not been well addressed so far.

The TRP superfamily has more than 20 members in mammals, most of which are ion channels with temperature‐sensitive functions expressed on neurons being not associated with epidermal development (Saito and Shingai, 2006). However, the transient receptor potential cation channel, subfamily V, member 3 (*TRPV3*), is known to be highly expressed in keratinocytes (Chung et al., 2004; Peier et al., 2002; Xu et al., 2002). Actually, *TRPV3* is not only a thermosensitive TRP channel but also related to epidermal development (Cheng et al., 2010). Humans with *TRPV3* mutations had abnormal keratinization disease. For example, a mutation of *TRPV3* or membrane‐bound transcription factor protease site 2 (*MBTPS2*) could lead to Olmsted syndrome, described by bilateral mutilating palmoplantar keratoderma (PPK) and periorificial keratotic plaques (Gatault et al., 2020). *TRPV3* regulated by mutated *MBTPS2* or directly mutated *TRPV3* might function in a dominant‐positive manner to increase constitutive *TRPV3* activity and elevate Ca^2+^ in keratinocytes, leading to severe keratoderma (Lin et al., 2012; Nemer et al., 2017). In addition, this gene could affect epidermal development by regulating the epidermal growth factor receptor (EGFR) signaling pathway (Cheng et al., 2010).

**FIGURE 1:**
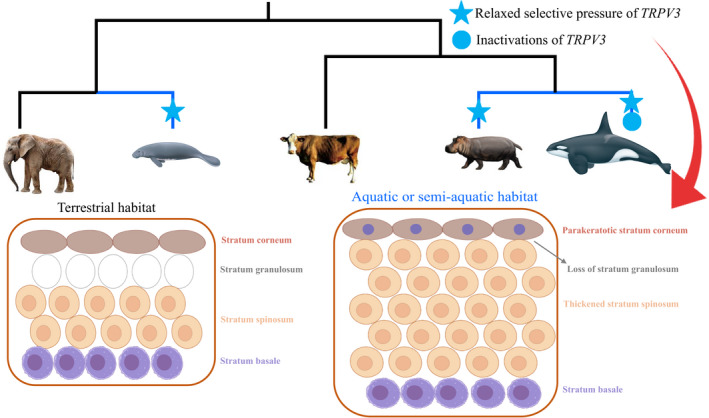
Inactivating mutations were detected in *TRPV3* of almost all the cetaceans. Relaxed selective constraints on the *TRPV3* gene were convergently detected in three clades (cetaceans, manatees, and hippopotamus) with similar epidermis structure, such as parakeratotic stratum corneum, loss of stratum granulosum, and thickened stratum spinosum. These results suggested that the decay of *TRPV3* might be the genomic trace of epidermal structure changes in the land‐to‐sea transition of mammals

A total of 142 mammalian *TRPV3* sequences were analyzed in this study to investigate the potential association between *TRPV3* gene evolution and phenotypic changes in the epidermal structure. Inactivating mutations were found in cetaceans and relaxed selection pressure was detected in some species with the analogously specialized epidermis (including cetaceans, manatees, and hippopotamus) (Figure 1). These findings could provide some novel insights into the evolutionary mechanisms underlying the epidermis evolution of mammals.

## 2 MATERIALS AND METHODS

### 2.1 Sequence mining and BLAST searches

The present study used 142 mammals (covering 4 superorders and 19 orders) with high‐quality genomes (Table S1). *TRPV3*‐coding DNA sequences (CDS) from National Center for Biotechnology Information (NCBI, https://www.ncbi.nlm.nih.gov/) for five representative species with annotated genomes, that is, human (*Homo sapiens*), mouse (*Mus musculus*), cow (*Bos taurus*), nine‐banded armadillo (*Dasypus novemcinctus*), and African elephant (*Loxodonta africana*), were first extracted. About 50‐bp flanking sequences of each exon from these species were used as reference sequences to BLAST against other mammals. The longest predicted version from the NCBI database was downloaded for species with low‐quality sequences from local BLAST. In addition, the *TRPV3* sequence of the bowhead whale (*Balaena mysticetus*) was downloaded from http://www.bowhead‐whale.org/. The sequences were aligned in MEGA7 with the MUSCLE (Edgar, 2004) plug‐in and minor adjustments by eye.

### 2.2 Identification of inactivating mutations

The existence of inactivation mutations was an interesting discovery during the comparative analysis of the sequences. *TRPV3* sequences of 142 mammals were inspected for inactivating mutations, including mutation of initiation codons and stop codons, frameshift insertions and deletions, premature stop codons, splice site mutation of intron/exon boundary [GT/AG], and so forth. In addition, the ancestral sequences were reconstructed using PRANK (Löytynoja, 2014) with the “‐showanc” parameter to test the presence of inactivating mutations in the sequence of cetacean ancestors.

Moreover, putative inactivating mutations were further verified by raw sequencing reads using the method described in Jebb and Hiller (2018). Briefly, the scaffolds containing a mutation were extracted; these sequences were aligned against sequencing reads stored in Sequence Read Archive (SRA) (Kodama et al., 2012), which included skin transcriptomes data, resequencing data, and raw sequencing data. After downloading the SRA data using SRAtoolkit, the original data were converted into FASTQ format files using the fastq software with “‐split‐3” parameter. A library of scaffolds with inactivated mutations and map reads was constructed using the Burrows–Wheeler Alignment Tool (BWA) (Li, 2013). The Samtools software was used to obtain the file in BAM format (Li et al., 2009). Finally, IGV software was used for sequence alignment visualization (Thorvaldsdóttir et al., 2013).

These inactivating mutations were further confirmed by polymerase chain reaction (PCR) amplification. Three types of mutations, including splice sites mutation, premature stop codons, and deletions, were separately amplified in three representative species of cetaceans, that is, baiji (*Lipotes vexillifer*, AG‐AA), common minke whale (*B*. *acutorostrata*, AAA‐TAA), and Yangtze finless porpoise (*Neophocaena asiaeorientalis*, 6‐bp deletion). Genomic DNA extraction, PCR amplification, and sequencing were conducted as described by S. Xu et al. (2012). The primers are listed in Table S2.

### 2.3 Phylogenetic analysis

A supermatrix approach was used by IQ‐TREE for the maximum‐likelihood (ML) tree inference of *TRPV3* (Nguyen et al., 2015). The ‐m MFP option that could cause the ModelFinder program to select the best model was used (Kalyaanamoorthy et al., 2017). Bootstrap analyses with 1000 pseudo‐replicates were conducted. All other options were set to their default values in IQ‐TREE.

### 2.4 Selection analyses

The nonsynonymous (*d*_N_)/synonymous substitution (*d*_S_) rate (*ω* = *d*_N_/*d*_S_) was used to evaluate the selective pressure, with values of *ω* > 1, =1, and <1 indicating positive, neutral, and purifying selection, respectively. The *ω* values were calculated using the codon‐based ML models implemented in the CODEML program of PAML 4.9e (Yang, 2007). The well‐acknowledged phylogenetic tree was downloaded from http://www.timetree.org/ as an input tree for PAML analysis.

The free‐ratio model that assumed an independent *ω* for each branch was compared with the null one‐ratio model that allowed the same *ω* for all branches to evaluate the selective pressure in different branches with intact *TRPV3*. The likelihood ratio test statistic approximated to a chi‐square distribution and was used to compare nested likelihood models. The average *ω* of each order or superorder was calculated according to the result of the free‐ratio model.

The branch model implemented in CODEML was used to test whether *TRPV3* was under relaxed selective pressure in species with inactivating mutations. Two different datasets were used in the study. All species with intact *TRPV3* but without specialization of the epidermal structure were included in both datasets: Dataset I including additional 12 cetaceans with inactivating mutations and dataset Ⅱ consisting of additional 6 terrestrial mammals with inactivating mutations. Further, the relaxed selection was also detected using the program RELAX (Wertheim et al., 2015), which computed the values and distribution of three *ω* using a branch‐site model, the convergence of the three *ω* values toward one in a lineage being a signature of relaxed purifying selection. The magnitude of convergence depended on a parameter K, which tended to zero as the selection tended to be completely relaxed.

### 2.5 Time estimations for gene inactivation

The method described by Chou et al. (2002) and Mu et al. (2021) was used to estimate when *TRPV3* was inactivated in lineages of cetaceans. This method presumed that the branches along which the genes became pseudogenes went through two periods. The gene that evolved under selective pressure (*ω_s_*) was similar to that in other species until it was inactivated. Then, this gene was presumed to accumulate both nonsynonymous and synonymous mutations at an equal rate (*ω_n_* = 1). Thus, the following equations were used:

$$\omega_{a}=\omega_{s}\times T_{s}/T+\omega_{n}\times T_{n}/T$$

$$T_{s}=T‐T_{n}$$

to calculate the *T_n_* value, where *T* is the time since the split from the last common ancestor. The lower and upper bounds of the confidence interval for the species divergence time *T* were obtained from http://www.timetree.org/. The *ω_a_* value was assessed for the entire branch.

## 3 RESULTS

### 3.1 Sequence alignment and phylogenetic analysis

**FIGURE 2:**
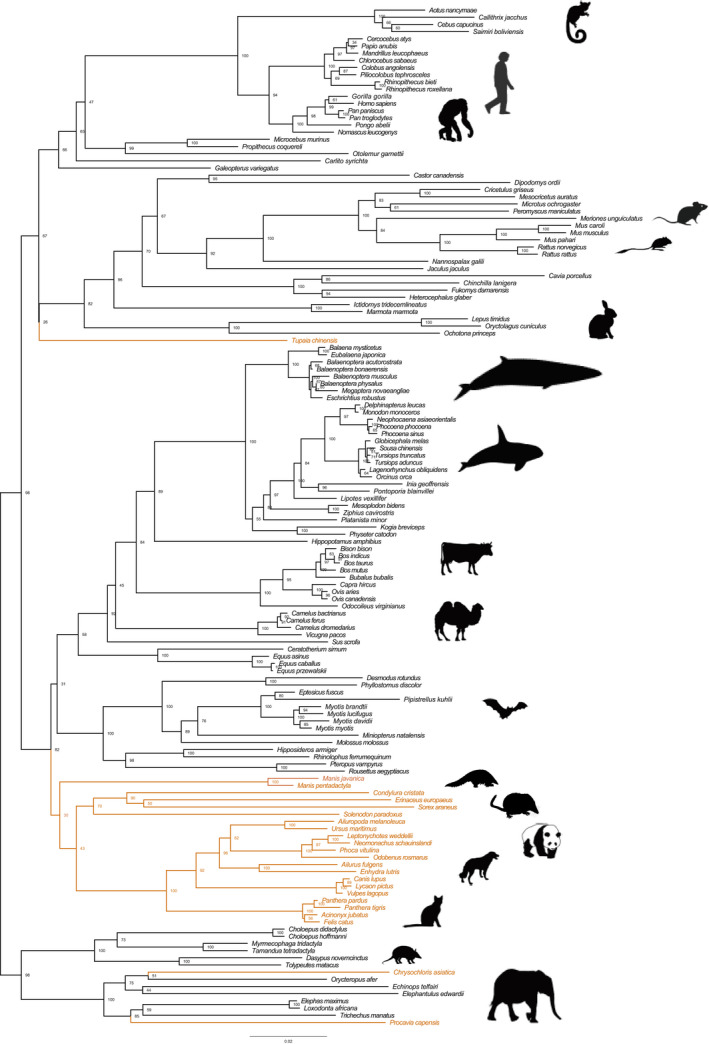
Phylogenetic tree of *TRPV3*. Using 142 mammalian *TRPV3* coding sequences as materials, the phylogenetic tree was reconstructed by the maximum‐likelihood (ML) method using IQ‐TREE. Inconsistencies were found in the clades highlighted in brown when comparing the species tree. The bootstrap values of 78% of nodes were >0.70, which suggested that the *TRPV3* gene tree was well supported

A total of 131 species examined in this study possessed complete *TRPV3* CDS and 11 species with incomplete sequences, but more than or approximately equal to 90% of CDS, except for the naked mole‐rat (*Heterocephalus glaber*) having 78% of CDS (Table S3). However, not all complete genes were intact; inactivating mutations were detected in most lineages of cetaceans and several terrestrial lineages. A phylogenetic tree of the *TRPV3* gene was reconstructed, with 78% of nodes having bootstrap values >0.70 (Figure 2). Almost all orders and superorders (Laurasiatheria, Euarchontoglires, Xenarthra, and Afrotheria) were consistent with the widely recognized phylogenetic tree, except for Afrosoricida (Figure 2). However, the phylogenetic relationship within several orders differed from the well‐accepted tree. For example, Scandentia was sister to the Primates + Dermoptera clade in the recognized phylogenetic tree but formed a sister group with the clade including Rodentia and Lagomorpha in the gene tree. Carnivora, Pholidota, and Eulipotyphla were clustered forming a clade, but the recognized tree was not.

### 3.2 Inactivating mutations

**FIGURE 3:**
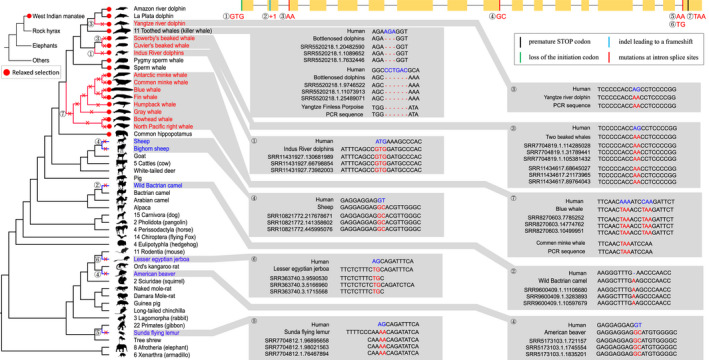
Inactivating mutations of *TRPV3* in multiple mammalian lineages. Left: Phylogeny of 142 placental mammals, in which red font represents species with inactivating mutations and under the relaxed selection pressure of *TRPV3*, while blue font represents species with inactivating mutations but not under the relaxed selection pressure of *TRPV3*. The red solid circle represents species (cetaceans, manatees, and hippopotamus) under the relaxed selection pressure of *TRPV3* in the placental mammal range. Right: The intact *TRPV3* gene model is visualized at the top, superimposed with multiple specific inactivated mutations. Different colors represent different types of mutations. The inactivating mutations were validated by SRA reads and PCR amplification reaction. The alignments of reads are highlighted with gray background, and each inactivating mutation corresponds to a serial number. Sequences in red font represent the mutated sites, while sequences in blue font represent the sequences of humans used as a reference. Notably, the deletions of 3 and 6 bp cover 11 toothed whales, including common bottlenose dolphin *Tursiops truncates*, Indo‐Pacific bottlenose dolphin *Tursiops aduncus*, Indo‐Pacific humpback dolphin *Sousa chinensis*, Pacific white‐sided dolphin *Lagenorhynchus obliquidens*, long‐finned pilot whale *Globicephala melas*, killer whale *Orcinus orca*, harbor porpoise *Phocoena phocoena*, vaquita *Phocoena sinus*, Yangtze finless porpoise, beluga whale *Delphinapterus leucas*, and narwhal *Monodon monoceros*

Inactivating mutations were identified in 18 species after assessing reliability by matching with available sequencing reads (Sharma et al., 2020) and further PCR (Figure 3 and Table S4). Premature stop codons were detected in eight baleen whales (fin whale *Balaenoptera physalus*, common minke whale, Antarctic minke whale *B*. *bonaerensis*, blue whale *B*. *musculus*, bowhead whale *Balaena mysticetus*, gray whale *Eschrichtius robustus*, North Pacific right whale *Eubalaena japonica*, and humpback whale *Megaptera novaeangliae*), with one premature stop codon sheared among these species. Through ancestral sequences reconstruction, *TRPV3* was found to be lost in the lineage of crown Mysticeti due to the shared premature stop codon. The initial codon mutation was detected in the blue whale (n. ATG → ATA, p. M → I) and Indus River dolphin (*Platanista minor*, n. ATG → GTG, p. M → V). Splice site mutations were found in three toothed whales (sowerby's beaked whale *Mesoplodon bidens*, cuvier's beaked whale *Ziphius cavirostris*, and baiji). No inactivating mutation was found in the most recent common ancestor of all cetaceans. Moreover, 3‐ and 6‐bp deletions were found to be shared by 11 toothed whales (Figure 3). The inactivating mutations of baiji and common minke whale and the deletions of the Yangtze finless porpoise *TRPV3* gene were verified using PCR. Splice site mutations were found in five terrestrial species (sheep *Ovis aries*, bighorn sheep *O*. *canadensis*, American beaver *Castor canadensis*, sunda flying lemur *Galeopterus variegatus*, and lesser Egyptian jerboa *Jaculus jaculus*), and frameshift indels were detected in wild Bactrian camel (*Camelus ferus*). For 11 incomplete *TRPV3* sequences, no signal of pseudogenization was detected.

### 3.3 Selective pressure detection on *TRPV3*

**FIGURE 4:**
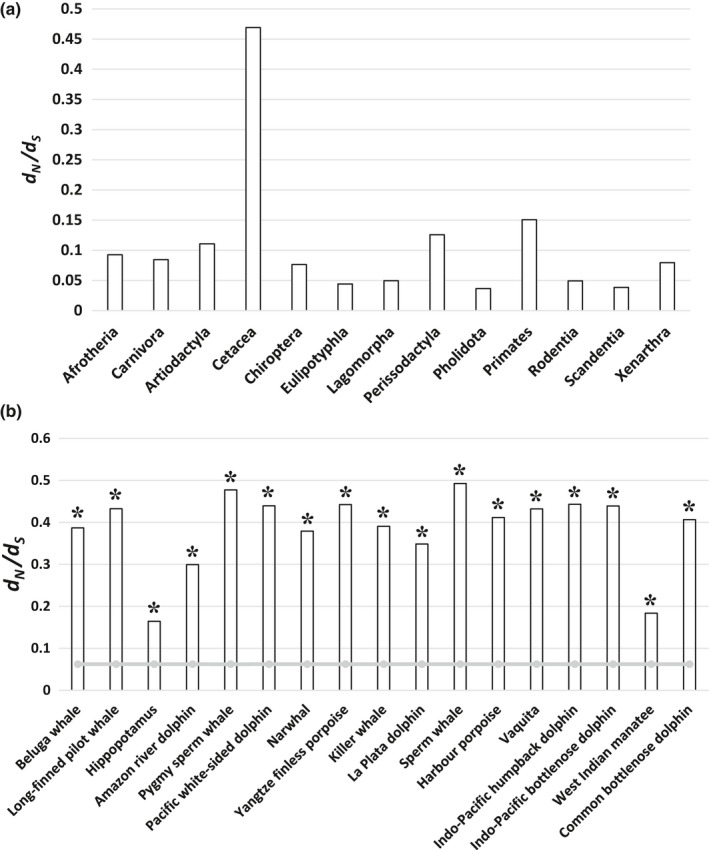
Results of the selection test. (a) Average evolution rate value (*ω*) of each branch in mammals. The *y*‐coordinate represents the value of *ω*. All *ω* values of order or superorder are averagely below 0.1 or around 0.1, except the *ω* of Cetacea near 0.5. Clearly, the amino acid substitution rate in cetaceans was much higher than that in other mammalian groups, suggesting the relaxed selection pressure of cetaceans. (b) Evolution rate value (*ω*) of species with intact *TRPV3* but changed epidermal structure. The *y*‐coordinate represents the value of *ω*. The *ω* of terrestrial mammals (background species) is represented by a gray curve. The *ω* of species with intact *TRPV3* but changed epidermal structure (tested species) is represented by a column. The asterisk represents a significantly improved *ω* value for the tested species compared with the *ω* of background species. This indicated that some aquatic or semi‐aquatic mammals (cetaceans, manatees, and hippopotamus) were under relaxed selection pressure, although their sequences of *TRPV3* were intact

The one‐ratio model revealed that the *ω* of mammals with intact *TRPV3* sequences was 0.07188. The likelihood ratio tests suggested that the free‐ratio model was better than the one‐ratio model (*p* < .001). The most average *ω* of order or superorder was below 0.1 or around 0.1 (Figure 4a). Interestingly, only the *ω* for Cetacea significantly increased (*ω* = .47); particularly, the *ω* of some whales was close to 1. This result suggested that selective pressure was relaxed in cetacean lineages, although they had an intact *TRPV3*.

Relaxed selective pressure was further detected in cetacean lineages with inactivating mutations (Table 1). In dataset I, the average *ω* across the tree was estimated to be 0.06834 in Model A, assuming that all branches had the same *ω*, which was significantly <1 (Model B), indicating strong purifying selection on *TRPV3* (*p* = 0; Table 1). Model C that allowed cetacean lineages with pseudogenized *TRPV3* having *ω*2 (*ω*2 = 0.25874), whereas others had *ω*1 (*ω*1 = 0.06586), was used to test the presence of relaxed selective pressure on the pseudogenized cetacean branches, which fit the data significantly better than Model A (*p* = 2.55e−15, Table 1). Next, Model C and Model D were compared, in which the pseudogenized branches were fixed at *ω*2 = 1. The result showed that the selective pressure on *TRPV3* was not completely relaxed in 12 pseudogenized cetaceans (*p* = 1.10e−14 in Model C vs D). Finally, Model E, which allowed different branches to have their own *ω*, significantly fitted the data compared with Model C (*p* = 0 in Model C vs E), indicating a divergence in the selective pressure across the tree. The relaxed selective pressure on cetacean pseudogenized lineages was further corroborated by the results of RELAX when cetaceans with inactivating mutations were used as test branches (*K* = 0.28, *p* < .0001, Figure S1). However, in dataset Ⅱ, relaxed selection pressure was not detected in six terrestrial mammals with inactivating mutations using PAML (*ω*1 = 0.06238, *ω*2 = 0.05961 in Model C, *p* = 7.71e−01) and RELAX (Figure S1).

**TABLE 1:** Likelihood ratio test of selection on the *TRPV3* gene with inactivating mutations

	Models	*ω* (*d*_N_/*d*_S_)	lnL^a^	np^b^	Models compared	2Δ (ln *L*)^c^	*p* value
Dataset I:	119 sequences (107 background species^d^ plus 12 cetaceans with inactivating mutations)						
	All branches have one *ω* (A)	0.06834	–39769.38	238			
	All branches have one *ω* = 1 (B)	1	–45248.02	237	B vs. A	10957.28	0
	Branches with pseudogenized *TRPV3* have *ω*2; others have *ω*1 (C)	*ω*1 = 0.06586 *ω*2 = 0.25874	–39738.10	239	A vs. C	62.56	2.55E−15
	Branches with pseudogenized *TRPV3* have *ω*2 = 1; others have *ω*1 (D)	*ω*1 = 0.06584 *ω*2 = 1	–39767.96	238	D vs. C	59.72	1.10E−14
	Each branch has its own *ω* (E)	Variable *ω* by branch	–39473.34	473	C vs. E	529.52	0
Dataset II:	113 sequences (107 background species and 6 terrestrial mammals with inactivating mutations)						
	All branches have one *ω* (A)	0.06227	–39175.69	226			
	All branches have one *ω* = 1 (B)	1	–44932.98	225	B vs. A	11514.58	0
	Branches with pseudogenized *TRPV3* have *ω*2; others have *ω*1 (C)	*ω*1 = 0.06238 *ω*2 = 0.05961	–39175.65	227	A vs. C	0.08	7.71E−01
	Branches with pseudogenized *TRPV3* have *ω*2 = 1; others have *ω*1 (D)	*ω*1 = 0.06179 *ω*2 = 1	–39383.37	226	D vs. C	415.44	0
	Each branch has its own *ω* (E)	Variable *ω* by branch	–38990.45	449	C vs. E	370.40	0

^a^ The natural logarithm of the likelihood value.

^b^ Number of parameters.

^c^ Twice the difference in ln*L* between the two models compared.

^d^ 107 background branches including species with intact *TRPV3* but without specialization of epidermal structures.

Finally, the selective pressure in three clades (cetaceans, hippopotamus, and manatees) with intact *TRPV3* that shared similar changes in the epidermal structure was detected. The result showed that all three clades had significantly higher *ω* value, suggesting the relaxation of selective constraints in the three clades (Figures 3 and 4b). Also, the RELAX analysis showed that lineages of cetaceans (*K* = 0.24, *p* < .0001), hippopotamus (*K* = 0.56, *p* < .0001), and manatees (*K* = 0.55, *p* < .0001) were under relaxed selection relative to reference lineages (species without inactivating mutations or specialized epidermal structures) after comparing the alternative model with the null model (Figure S1).

### 3.4 *TRPV3* inactivation date

It was estimated that the *TRPV3* gene was inactivated 5.4–4.2 million years ago (Ma) in the baiji and 9.6–8.0 Ma in the Indus River dolphin. The mean estimate for the inactivating time of *TRPV3* on the beaked whale clade was 8.0–6.6 Ma. For baleen whales (Mysticeti), the inactivation on the ancestral lineage dated from 34 to 25.9 Ma according to the TimeTree website.

## 4 DISCUSSION

The skin is the first barrier in mammals. Phenotypic changes in the skin have been found in lineages inhabiting different habitats. For an extreme example, aquatic or semi‐aquatic mammals have evolved extremely thickened skin to deal with the complicated external environment. This study found that the *TRPV3* gene, which was related to the regulation of epidermal development, was inactivated in multiple lineages of mammals and was under relaxed selective pressure in all aquatic or semi‐aquatic mammals. In contrast, it was under purifying selection in other groups of terrestrial mammals. Signals of evolution analysis were reflected in species with specialized epidermal structures rather than in other phenotypes, such as temperature sensing of the nervous system. The molecular decay of *TRPV3*, therefore, could be regarded as the genomic trace corresponding to the epidermis structure evolution. Although some terrestrial mammals, such as elephants and pangolins, also have thicker skin structures, their thick epidermis is due to the thickening of the stratum corneum. Consistently, no similar molecular bases were found between them and cetaceans, at least in *TRPV3*.

### 4.1 Association between inactivated *TRPV3* and epidermal specialization in cetaceans

Previous anatomical studies suggested that cetaceans evolved the exceptionally thickened stratum spinosum, the lack of stratum granulosum, and the parakeratotic stratum corneum to adapt to a completely aquatic environment (Reeb et al., 2007; Spearman, 1972; Springer et al., 2021). This study found that the *TRPV3* gene was inactivated in almost all the cetaceans, including baleen whales and several toothed whales. Cetacean *TRPV3* genes were detected to have higher *ω* values, suggesting a relaxed selective pressure. *TRPV3* participates in keratinocyte differentiation by forming a signaling complex with *TGF*‐a/EGFR (Cheng et al., 2010), which is known to have at least two distinct functions in the epidermis: promoting keratinocyte proliferation in the basal layer (Schneider et al., 2008) and promoting late terminal differentiation in suprabasal cells (Ballarò et al., 2005; Wakita & Takigawa, 1999). *TRPV3* knock‐out mice exhibited a significant increase in the thickness of the stratum spinosum, and defective stratum granulosum and stratum corneum (Cheng et al., 2010), which perfectly matched with the epidermal phenotype of cetaceans. Therefore, the present finding of molecular decay in *TRPV3* might contribute to the specialization of epidermal structures in cetaceans in response to the completely aquatic environment. The cost of the functional loss of sensing warm temperatures of *TRPV3* could be compensated by other genes such as *TRPV4*. After all, a phenotype is usually not determined by a single gene.

### 4.2 Convergence in relaxed selection pressure of *TRPV3* in different aquatic or semi‐aquatic mammals

During the mammalian evolution, several deeply diverged lineages, such as cetaceans, manatees, and hippopotamus, independently returned to the aquatic environment. These lineages have convergent increased thickness of stratum spinosum to cope with increased difficulties in wound healing and different pathogenic environments. In addition, stratum granulosum was lost in cetaceans and manatees and ill‐defined in hippopotamus (Jarrett et al., 1965; Luck & Wright, 1964; Spearman, 1972). Further, a distinctive parakeratotic stratum corneum with flattened cells was found in cetaceans, providing protection from water penetration.

The results of this study revealed that *TRPV3* was inactivated in almost all the cetaceans, whereas it was intact in manatees and hippopotamuses. However, all three clades revealed relaxed selection pressure in this gene. In the cases of manatees and hippopotamuses, the accumulation of inactivating mutations might have lagged behind the relaxed selective pressure. This was similar to *MC5R*, a gene regulating the development of sebaceous glands, which was intact but under relaxed selection pressure in the hippopotamus and naked mole‐rat (*Heterocephalus glaber*). However, the relaxed selection pressure on *MC5R* was interpreted as a proxy for the loss of sebaceous glands in both species (Springer & Gatesy, 2018). The molecular convergence in the relaxed selection on *TRPV3* in different aquatic or semi‐aquatic mammals suggested that they might have evolved similar mechanisms driving the independent specialization of the epidermal structure in different mammalian lineages.

### 4.3 Functional adaptation of terrestrial mammals with inactivated *TRPV3*

During the mammalian evolution, inactivated mutations in *TRPV3* were also examined in six terrestrial mammals. However, selective pressure was not relaxed in these six lineages. In humans, splice site mutations could cause some genetic diseases, suggesting that this mutation pattern could result in functional changes (López‐Bigas et al., 2005). Thus, a splicing site mutation shared by sheep and bighorn sheep might be related to the incomplete cytolysis in the horny layers (Jarrett et al., 1965) and result in the epidermis only two or three cells thick (Lyne, 1957; Parakkal et al., 1962). Moreover, in both species, wool probably provided better protection against the cold than the normal skin of animals living at high altitudes. In American beavers, the splice site mutation of *TRPV3* might be associated with their scaly stratum corneum in the tail (Jenkins, & Busher, 1979). In addition, inactivating mutations were identified in the sequences of the lesser Egyptian jerboa (splice site mutation), sunda flying lemur (splice site mutation), and wild Bactrian camel (1‐bp insertion), all of which inhabited hot stressful environments. These mutations might be related to the tolerance against high temperatures because of the thermosensation function of *TRPV3* (Moqrich et al., 2005; Saito et al., 2011).

## 5 CONCLUSIONS

In this study, a comprehensive comparative analysis of mammalian *TRPV3* gene evolution was conducted, and its association with the diversity of epidermal developments in mammals was highlighted. Inactivated mutations and relaxed selective pressure on *TRPV3* were examined in almost all the cetacean lineages, which might contribute to its exceptionally thickened stratum spinosum in response to the completely aquatic environment. Relaxed selective pressure was also detected in aquatic manatees and semi‐aquatic hippopotamuses, although both clades had intact *TRPV3*. Therefore, a similar mechanism driving the independent specialization of the epidermal structure in the three clades to adapt to the aquatic environment was suggested. The study added *TRPV3* to the epidermal specialization‐related gene pool and provided some new insights into mammalian epidermal evolution. The findings will be verified in the future.

## CONFLICT OF INTEREST

The authors declare no conflicts of interest.

## AUTHOR CONTRIBUTIONS

**Tianzhen Wu:** Conceptualization (equal); Data curation (lead); Formal analysis (lead); Investigation (lead); Validation (lead); Visualization (lead); Writing – original draft (lead). **Luoying Deme:** Validation (equal). **Zhenhua Zhang:** Validation (equal). **Xin Huang:** Formal analysis (equal). **Shixia Xu:** Conceptualization (equal); Formal analysis (equal); Project administration (equal); Writing – review & editing (equal). **Guang Yang:** Conceptualization (equal); Data curation (equal); Formal analysis (equal); Funding acquisition (equal); Project administration (equal); Writing – review & editing (equal).

## Supporting information

Appendix S1

## Data Availability

All data supporting this study are provided as Appendix S1 accompanying this manuscript.
