# Supplementary material for: Decay of TRPV3 as the genomic trace of epidermal structure changes in the land‐to‐sea transition of mammals
Source: Ecol Evol. 2022 Mar 18;12(3):e8731. doi: 10.1002/ece3.8731 (PMC8931706; doi:10.1002/ece3.8731)

**Supplementary material**

**Table S1.** Information on 142 species selected for this article.

**Table S2.** The primers set of baiji, common minke whale and Yangtze finless porpoise.

**Table S3.** Missing length and proportion for 11 species with incomplete sequences.

**Table S4.** The accession numbers for each species with inactivated *TPPV3* and the reliability of the inactivating mutations by aligned with reads. Noticeably, the 3bp and 6bp deletions shared in 11 toothed whales were also verified.

**Figure S1.** The result of RELAX when respectively regarded cetaceans with inactivating mutations, cetaceans without inactivating mutations, manatees, hippopotamus, sheep, bighorn sheep, lesser Egyptian jerboa, wild Bactrian camel, American beavers and Sunda flying lemur as test branches.

**Table S1**

| Superorder | Order | Latin name | Accession of RefSeq assembly or GenBank assembly |
| --- | --- | --- | --- |
| Euarchontoglires | Primates | *Homo sapiens* | GCF_000001405.39 |
| Euarchontoglires | Primates | *Pan troglodytes* | GCF_002880755.1 |
| Euarchontoglires | Primates | *Pan paniscus* | GCF_013052645.1 |
| Euarchontoglires | Primates | *Gorilla gorilla gorilla* | GCF_008122165.1 |
| Euarchontoglires | Primates | *Pongo pygmaeus abelii* | GCF_002880775.1 |
| Euarchontoglires | Primates | *Nomascus leucogenys* | GCF_006542625.1 |
| Euarchontoglires | Primates | *Papio anubis* | GCF_008728515.1 |
| Euarchontoglires | Primates | *Mandrillus leucophaeus* | GCF_000951045.1 |
| Euarchontoglires | Primates | *Cercocebus atys* | GCF_000955945.1 |
| Euarchontoglires | Primates | *Chlorocebus sabaeus* | GCF_000409795.2 |
| Euarchontoglires | Primates | *Rhinopithecus roxellana* | GCF_007565055.1 |
| Euarchontoglires | Primates | *Rhinopithecus bieti* | GCF_001698545.1 |
| Euarchontoglires | Primates | *Colobus angolensis palliatus* | GCF_000951035.1 |
| Euarchontoglires | Primates | *Piliocolobus tephrosceles* | GCF_002776525.3 |
| Euarchontoglires | Primates | *Callithrix jacchus* | GCF_009663435.1 |
| Euarchontoglires | Primates | *Aotus nancymaae* | GCF_000952055.2 |
| Euarchontoglires | Primates | *Saimiri boliviensis* | GCF_000235385.1 |
| Euarchontoglires | Primates | *Cebus capucinus imitator* | GCF_001604975.1 |
| Euarchontoglires | Primates | *Carlito syrichta* | GCF_000164805.1 |
| Euarchontoglires | Primates | *Otolemur garnettii* | GCF_000181295.1 |
| Euarchontoglires | Primates | *Microcebus murinus* | GCF_000165445.2 |
| Euarchontoglires | Primates | *Propithecus coquereli* | GCF_000956105.1 |
| Euarchontoglires | Dermoptera | *Galeopterus variegatus* | GCF_000696425.1 |
| Euarchontoglires | Scandentia | *Tupaia chinensis* | GCF_000334495.1 |
| Euarchontoglires | Rodentia | *Jaculus jaculus* | GCF_000280705.1 |
| Euarchontoglires | Rodentia | *Microtus ochrogaster* | GCF_000317375.1 |
| Euarchontoglires | Rodentia | *Cricetulus griseus* | GCF_000223135.1 |
| Euarchontoglires | Rodentia | *Mesocricetus auratus* | GCF_000349665.1 |
| Euarchontoglires | Rodentia | *Peromyscus maniculatus bairdii* | GCF_000500345.1 |
| Euarchontoglires | Rodentia | *Mus musculus* | GCF_000001635.27 |
| Euarchontoglires | Rodentia | *Mus caroli* | GCF_900094665.1 |
| Euarchontoglires | Rodentia | *Mus pahari* | GCF_900095145.1 |
| Euarchontoglires | Rodentia | *Rattus norvegicus* | GCF_000001895.5 |
| Euarchontoglires | Rodentia | *Meriones unguiculatus* | GCF_002204375.1 |
| Euarchontoglires | Rodentia | *Nannospalax galili* | GCF_000622305.1 |
| Euarchontoglires | Rodentia | *Castor canadensis* | GCF_001984765.1 |
| Euarchontoglires | Rodentia | *Dipodomys ordii* | GCF_000151885.1 |
| Euarchontoglires | Rodentia | *Rattus rattus* | GCF_011064425.1 |
| Euarchontoglires | Rodentia | *Heterocephalus glaber* | GCF_000247695.1 |
| Euarchontoglires | Rodentia | *Fukomys damarensis* | GCF_012274545.1 |
| Euarchontoglires | Rodentia | *Cavia porcellus* | GCF_000151735.1 |
| Euarchontoglires | Rodentia | *Chinchilla lanigera* | GCF_000276665.1 |
| Euarchontoglires | Rodentia | *Ictidomys tridecemlineatus* | GCF_000236235.1 |
| Euarchontoglires | Rodentia | *Marmota marmota* | GCF_001458135.1 |
| Euarchontoglires | Lagomorpha | *lepus timidus* | GCA_009760805.1 |
| Euarchontoglires | Lagomorpha | *Oryctolagus cuniculus* | GCF_000003625.3 |
| Euarchontoglires | Lagomorpha | *Ochotona princeps* | GCF_000292845.1 |
| Laurasiatheria | Cetartiodactyla | *Vicugna pacos* | GCF_000164845.3 |
| Laurasiatheria | Cetartiodactyla | *Camelus ferus* | GCF_009834535.1 |
| Laurasiatheria | Cetartiodactyla | *Camelus bactrianus* | GCF_000767855.1 |
| Laurasiatheria | Cetartiodactyla | *Camelus dromedarius* | GCF_000803125.2 |
| Laurasiatheria | Cetartiodactyla | *Tursiops truncatus* | GCF_011762595.1 |
| Laurasiatheria | Cetartiodactyla | *Orcinus orca* | GCF_000331955.2 |
| Laurasiatheria | Cetartiodactyla | *Delphinapterus leucas* | GCF_002288925.2 |
| Laurasiatheria | Cetartiodactyla | *Lipotes vexillifer* | GCF_000442215.1 |
| Laurasiatheria | Cetartiodactyla | *Physeter catodon* | GCF_002837175.2 |
| Laurasiatheria | Cetartiodactyla | *Balaenoptera physalus* | GCA_008795845.1 |
| Laurasiatheria | Cetartiodactyla | *Balaenoptera acutorostrata* | GCF_000493695.1 |
| Laurasiatheria | Cetartiodactyla | *Balaena mysticetus* | http://www.bowhead-whale.org/ |
| Laurasiatheria | Cetartiodactyla | *Megaptera novaeangliae* | GCA_004329385.1 |
| Laurasiatheria | Cetartiodactyla | *Balaenoptera bonaerensis* | GCA_000978805.1 |
| Laurasiatheria | Cetartiodactyla | *Balaenoptera musculus* | GCA_009873245.2 |
| Laurasiatheria | Cetartiodactyla | *Eschrichtius robustus* | GCA_002189225.1 |
| Laurasiatheria | Cetartiodactyla | *Eubalaena japonica* | GCA_004363455.1 |
| Laurasiatheria | Cetartiodactyla | *Globicephala melas* | GCA_006547405.1 |
| Laurasiatheria | Cetartiodactyla | *Inia geoffrensis* | GCA_004363515.1 |
| Laurasiatheria | Cetartiodactyla | *Kogia breviceps* | GCA_004363705.1 |
| Laurasiatheria | Cetartiodactyla | *Lagenorhynchus obliquidens* | GCF_003676395.1 |
| Laurasiatheria | Cetartiodactyla | *Mesoplodon bidens* | GCA_004027085.1 |
| Laurasiatheria | Cetartiodactyla | *Monodon monoceros* | GCF_005190385.1 |
| Laurasiatheria | Cetartiodactyla | *Neophocaena asiaeorientalis* | GCF_003031525.1 |
| Laurasiatheria | Cetartiodactyla | *Phocoena sinus* | GCF_008692025.1 |
| Laurasiatheria | Cetartiodactyla | *Platanista minor* | GCA_004363435.1 |
| Laurasiatheria | Cetartiodactyla | *Pontoporia blainvillei* | GCA_011754075.1 |
| Laurasiatheria | Cetartiodactyla | *Sousa chinensis* | GCA_007760645.1 |
| Laurasiatheria | Cetartiodactyla | *Tursiops aduncus* | GCA_003227395.1 |
| Laurasiatheria | Cetartiodactyla | *Ziphius cavirostris* | GCA_004364475.1 |
| Laurasiatheria | Cetartiodactyla | *Phocoena phocoena* | GCA_003071005.1 |
| Laurasiatheria | Cetartiodactyla | *Hippopotamus amphibius* | GCA_004027065.2 |
| Laurasiatheria | Cetartiodactyla | *Bos taurus* | GCF_002263795.1 |
| Laurasiatheria | Cetartiodactyla | *Bos indicus* | GCF_000247795.1 |
| Laurasiatheria | Cetartiodactyla | *Bison bison bison* | GCF_000754665.1 |
| Laurasiatheria | Cetartiodactyla | *Bos grunniens mutus* | GCF_000298355.1 |
| Laurasiatheria | Cetartiodactyla | *Bubalus bubalis* | GCF_003121395.1 |
| Laurasiatheria | Cetartiodactyla | *Ovis aries* | GCF_002742125.1 |
| Laurasiatheria | Cetartiodactyla | *Ovis canadensis canadensis* | GCA_004026945.1 |
| Laurasiatheria | Cetartiodactyla | *Capra hircus* | GCF_001704415.1 |
| Laurasiatheria | Cetartiodactyla | *Odocoileus virginianus texanus* | GCF_002102435.1 |
| Laurasiatheria | Cetartiodactyla | *Sus scrofa* | GCF_000003025.6 |
| Laurasiatheria | Perissodactyla | *Equus caballus* | GCF_002863925.1 |
| Laurasiatheria | Perissodactyla | *Equus przewalskii* | GCF_000696695.1 |
| Laurasiatheria | Perissodactyla | *Equus asinus* | GCF_001305755.1 |
| Laurasiatheria | Perissodactyla | *Ceratotherium simum* | GCF_000283155.1 |
| Laurasiatheria | Carnivora | *Felis catus* | GCF_000181335.3 |
| Laurasiatheria | Carnivora | *Acinonyx jubatus* | GCF_003709585.1 |
| Laurasiatheria | Carnivora | *Panthera tigris altaica* | GCF_000464555.1 |
| Laurasiatheria | Carnivora | *Panthera pardus* | GCF_001857705.1 |
| Laurasiatheria | Carnivora | *Canis lupus familiaris* | GCF_000002285.3 |
| Laurasiatheria | Carnivora | *Lycaon pictus* | GCA_001887905.1 |
| Laurasiatheria | Carnivora | *Enhydra lutris kenyoni* | GCF_002288905.1 |
| Laurasiatheria | Carnivora | *Ailurus fulgens styani* | GCA_002007465.1 |
| Laurasiatheria | Carnivora | *Ailuropoda melanoleuca* | GCF_002007445.1 |
| Laurasiatheria | Carnivora | *Vulpes lagopus* | GCA_004023825.1 |
| Laurasiatheria | Carnivora | *Ursus maritimus* | GCF_000687225.1 |
| Laurasiatheria | Carnivora | *Odobenus rosmarus divergens* | GCF_000321225.1 |
| Laurasiatheria | Carnivora | *Phoca vitulina* | GCA_004348235.1 |
| Laurasiatheria | Carnivora | *Leptonychotes weddellii* | GCF_000349705.1 |
| Laurasiatheria | Carnivora | *Neomonachus schauinslandi* | GCF_002201575.1 |
| Laurasiatheria | Pholidota | *Manis pentadactyla* | GCA_014570555.1 |
| Laurasiatheria | Pholidota | *Manis javanica* | GCF_001685135.1 |
| Laurasiatheria | Chiroptera | *Rhinolophus ferrumequinum* | GCF_004115265.1 |
| Laurasiatheria | Chiroptera | *Pteropus vampyrus* | GCF_000151845.1 |
| Laurasiatheria | Chiroptera | *Myotis myotis* | GCA_014108235.1 |
| Laurasiatheria | Chiroptera | *Pipistrellus kuhlii* | GCA_014108245.1 |
| Laurasiatheria | Chiroptera | *Molossus molossus* | GCA_014108415.1 |
| Laurasiatheria | Chiroptera | *Phyllostomus discolor* | GCF_004126475.2 |
| Laurasiatheria | Chiroptera | *Rousettus aegyptiacus* | GCF_001466805.2 |
| Laurasiatheria | Chiroptera | *Hipposideros armiger* | GCF_001890085.1 |
| Laurasiatheria | Chiroptera | *Eptesicus fuscus* | GCF_000308155.1 |
| Laurasiatheria | Chiroptera | *Myotis davidii* | GCF_000327345.1 |
| Laurasiatheria | Chiroptera | *Myotis brandtii* | GCF_000412655.1 |
| Laurasiatheria | Chiroptera | *Myotis lucifugus* | GCF_000147115.1 |
| Laurasiatheria | Chiroptera | *Miniopterus natalensis* | GCF_001595765.1 |
| Laurasiatheria | Chiroptera | *Desmodus rotundus* | GCF_002940915.1 |
| Laurasiatheria | Eulipotyphla | *Erinaceus europaeus* | GCF_000296755.1 |
| Laurasiatheria | Eulipotyphla | *Sorex araneus* | GCF_000181275.1 |
| Laurasiatheria | Eulipotyphla | *Solenodon paradoxus* | GCA_004363575.1 |
| Laurasiatheria | Eulipotyphla | *Condylura cristata* | GCF_000260355.1 |
| Afrotheria | Proboscidea | *Loxodonta africana* | GCF_000001905.1 |
| Afrotheria | Proboscidea | *Elephas maximus* | GCA_014332765.1 |
| Afrotheria | Sirenia | *Trichechus manatus latirostris* | GCF_000243295.1 |
| Afrotheria | Hyracoidea | *Procavia capensis* | GCA_004026925.2 |
| Afrotheria | Afrosoricida | *Chrysochloris asiatica* | GCF_000296735.1 |
| Afrotheria | Afrosoricida | *Echinops telfairi* | GCF_000313985.2 |
| Afrotheria | Macroscelidea | *Elephantulus edwardii* | GCF_000299155.1 |
| Afrotheria | Tubulidentata | *Orycteropus afer afer* | GCF_000298275.1 |
| Xenarthra | Cingulata | *Dasypus novemcinctus* | GCF_000208655.1 |
| Xenarthra | Cingulata | *Tolypeutes matacus* | GCA_004025125.1 |
| Xenarthra | Pilosa | *Tamandua tetradactyla* | GCA_004025105.1 |
| Xenarthra | Pilosa | *Myrmecophaga tridactyla* | GCA_004026745.1 |
| Xenarthra | Pilosa | *Choloepus didactylus* | GCA_004027855.1 |
| Xenarthra | Pilosa | *Choloepus hoffmanni* | GCA_000164785.2 |

**Table S2**

| The mutation sites and short stretches of flanking regions about 150bp for three cetaceans were amplified and sequenced using the following forward (F) and reverse (R) primers (5′ to 3′). |
| --- |
| Baiji-F: TGTCACCAAGACCTCTCCCCTCATCTTCTCCAAGC |
| Baiji-R: ACAGTATGCAGCAGAATGAGAACTTAACGCAATGCC |
| Common minke whale-F: CTTCTAAGGCAGGTGGTGGAC |
| Common minke whale-R: GGGTGCTTTCCTCGTCTCTA |
| Yangtze finless porpoise-F: CGCACAATTCTGGGTTTCCAC |
| Yangtze finless porpoise-R: AAATGCCTCCCCAAAGTCTCC |

**Table S3**

| Species | Missing length | Missing proportion | CDS proportion |
| --- | --- | --- | --- |
| *Balaenoptera_physalus* | 228 | 9.60% | 90.40% |
| *Colobus_angolensis* | 242 | 10.19% | 89.81% |
| *Equus_przewalskii* | 105 | 4.42% | 95.58% |
| *Hipposideros_armiger* | 529 | 22.26% | 77.74% |
| *Marmota_marmota* | 177 | 7.45% | 92.55% |
| *Mus_pahari* | 141 | 5.93% | 94.07% |
| *Myotis_lucifugus* | 193 | 8.12% | 91.88% |
| *Oryctolagus_cuniculus* | 186 | 7.83% | 92.17% |
| *Panthera_tigris* | 66 | 2.78% | 97.22% |
| *Pteropus_vampyrus* | 102 | 4.29% | 95.71% |
| *Trichechus_manatus* | 282 | 11.87% | 88.13% |

**Table S4**

| **species** | **mutation type** | **accessions** | **type of SRA date** | **result of verification** |
| --- | --- | --- | --- | --- |
| *Balaenoptera physalus* | shared premature stop codon |  |  | TRUE |
| *Balaena mysticetus* | shared premature stop codon |  |  | TRUE |
| *Balaenoptera acutorostrata* | shared premature stop codon | Verificated by PCR |  | TRUE |
| *Balaenoptera bonaerensis* | shared premature stop codon |  |  | TRUE |
| *Balaenoptera musculus* | shared premature stop codon, initial codon mutation | SRR8270603 | transcriptomes data | TRUE |
| *Eschrichtius robustus* | shared premature stop codon |  |  | TRUE |
| *Eubalaena japonica* | shared premature stop codon |  |  | TRUE |
| *Megaptera novaeangliae* | shared premature stop codon |  |  | TRUE |
| *Platanista minor* | initial codon mutation | SRR11431927 | raw sequencing data | TRUE |
| *Mesoplodon bidens* | shared splice site mutation | SRR7704819 | raw sequencing data | TRUE |
| *Lipotes vexillifer* | shared splice site mutation | Verificated by PCR |  | TRUE |
| *Ziphius cavirostris* | shared splice site mutation | SRR11434617 | raw sequencing data | TRUE |
| *Pontoporia blainvillei* | splice site mutation | SRR11431900 | resequencing data | FALSE |
| *Ovias aries* | shared splice site mutation |  |  | TRUE |
| *Ovis canadensis* | shared splice site mutation | SRR11431894 | raw sequencing data | TRUE |
| *Castor canadensis* | splice site mutation | SRR5173103 | raw sequencing data | TRUE |
| *Galeopterus variegatus* | splice site mutation | SRR7704812 | resequencing data | TRUE |
| *Jaculus jaculus* | splice site mutation | SRR363740 | raw sequencing data | TRUE |
| *Loxodonta africana* | frameshift indels | SRR12799664 | resequencing data | FALSE |
| *Camelus ferus* | frameshift indels | SRR9600409 | raw sequencing data | TRUE |
| *Myotis davidii* | frameshift indels | SRR534559 | raw sequencing data | FALSE |
| *Delphinapterus leucas* | shared 3bp deletion and 6bp deletion |  |  | TRUE |
| *Globicephala melas* | shared 3bp deletion and 6bp deletion |  |  | TRUE |
| *Lagenorhynchus obliquidens* | shared 3bp deletion and 6bp deletion |  |  | TRUE |
| *Monodon monoceros* | shared 3bp deletion and 6bp deletion |  |  | TRUE |
| *Neophocaena asiaeorientalis* | shared 3bp deletion and 6bp deletion | Verificated by PCR |  | TRUE |
| *Orcinus orca* | shared 3bp deletion and 6bp deletion |  |  | TRUE |
| *Phocoena phocoena* | shared 3bp deletion and 6bp deletion |  |  | TRUE |
| *Phocoena sinus* | shared 3bp deletion and 6bp deletion |  |  | TRUE |
| *Sousa chinensis* | shared 3bp deletion and 6bp deletion |  |  | TRUE |
| *Tursiops aduncus* | shared 3bp deletion and 6bp deletion |  |  | TRUE |
| *Tursiops truncatus* | shared 3bp deletion and 6bp deletion | SRR5520218 | transcriptomes data | TRUE |

**Fig. S1**

**
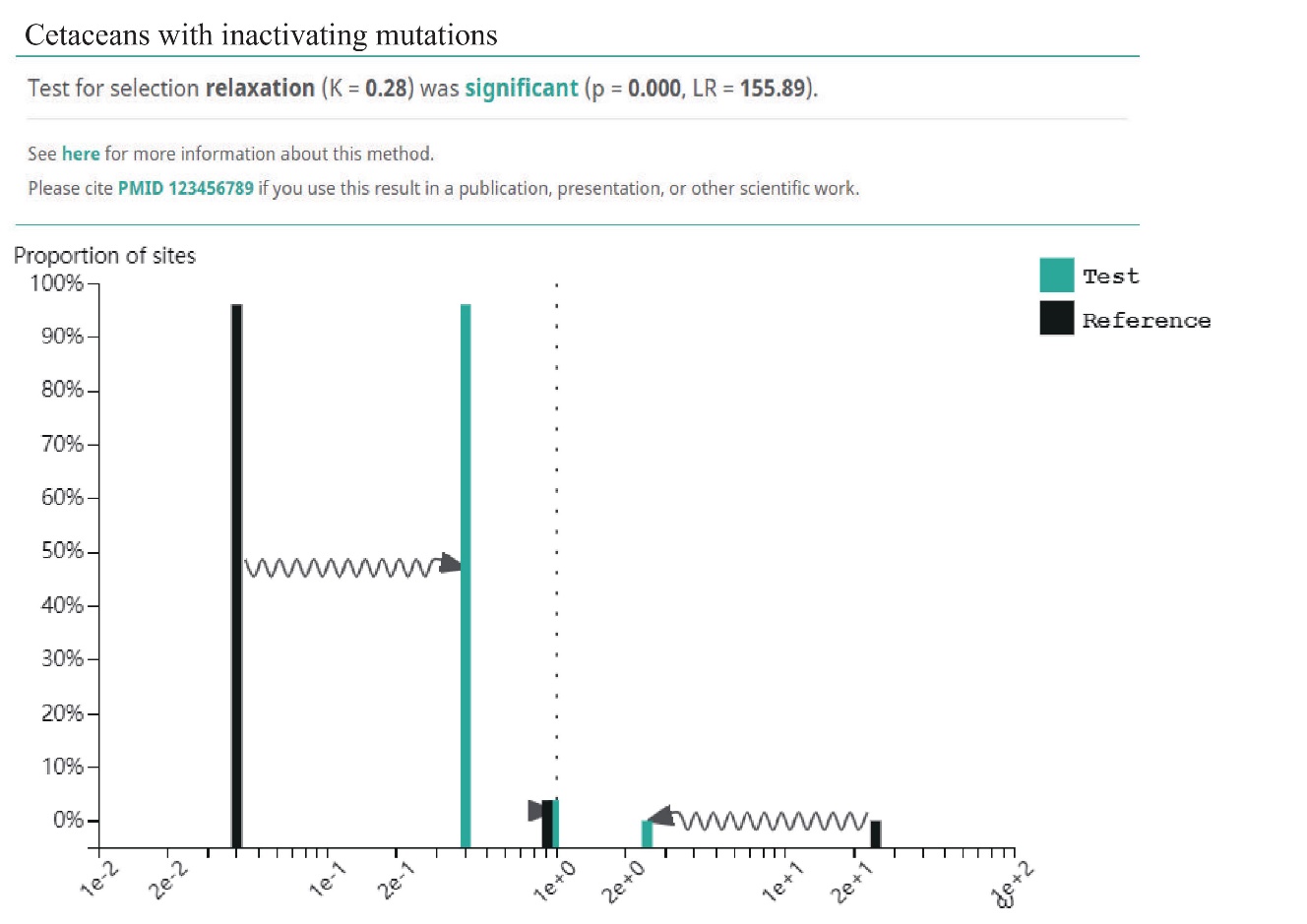
**
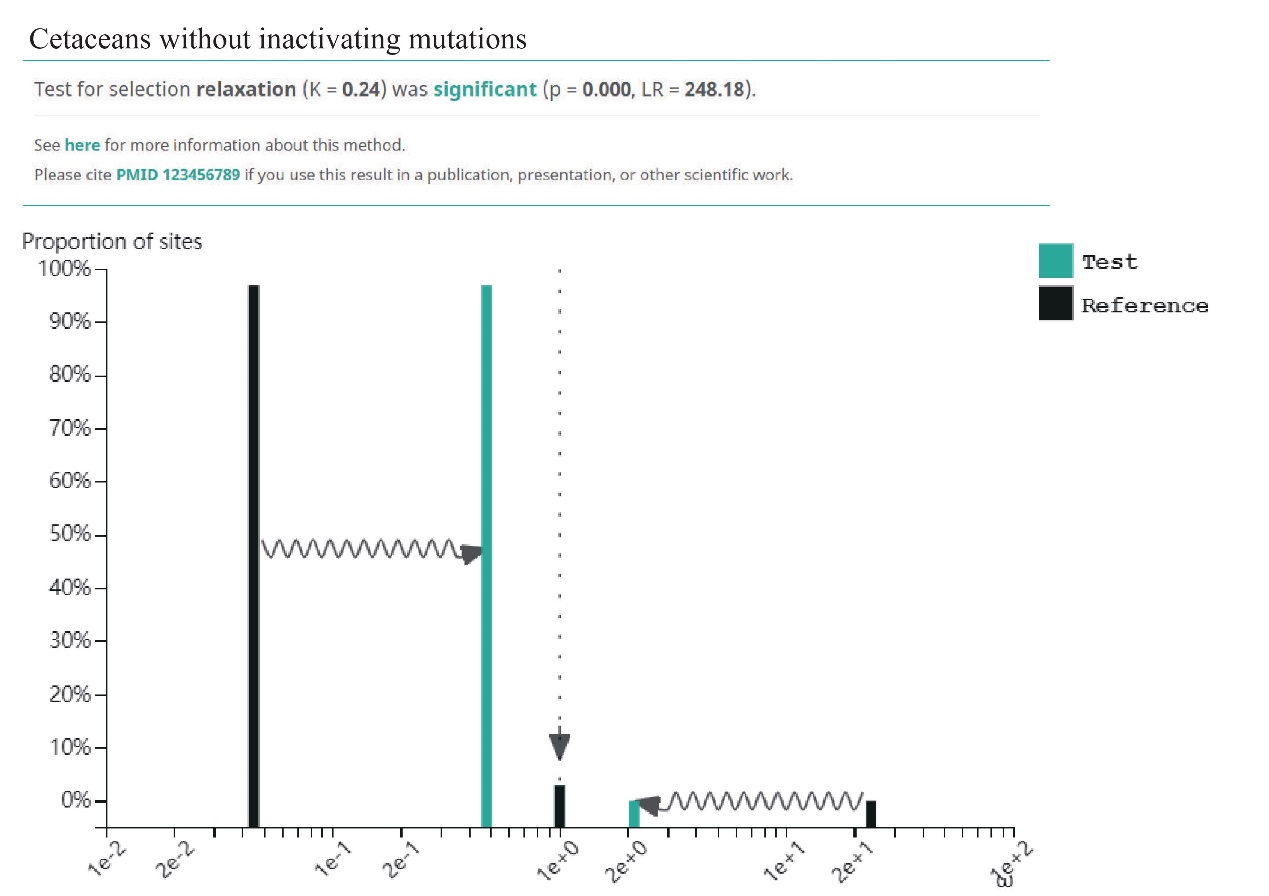


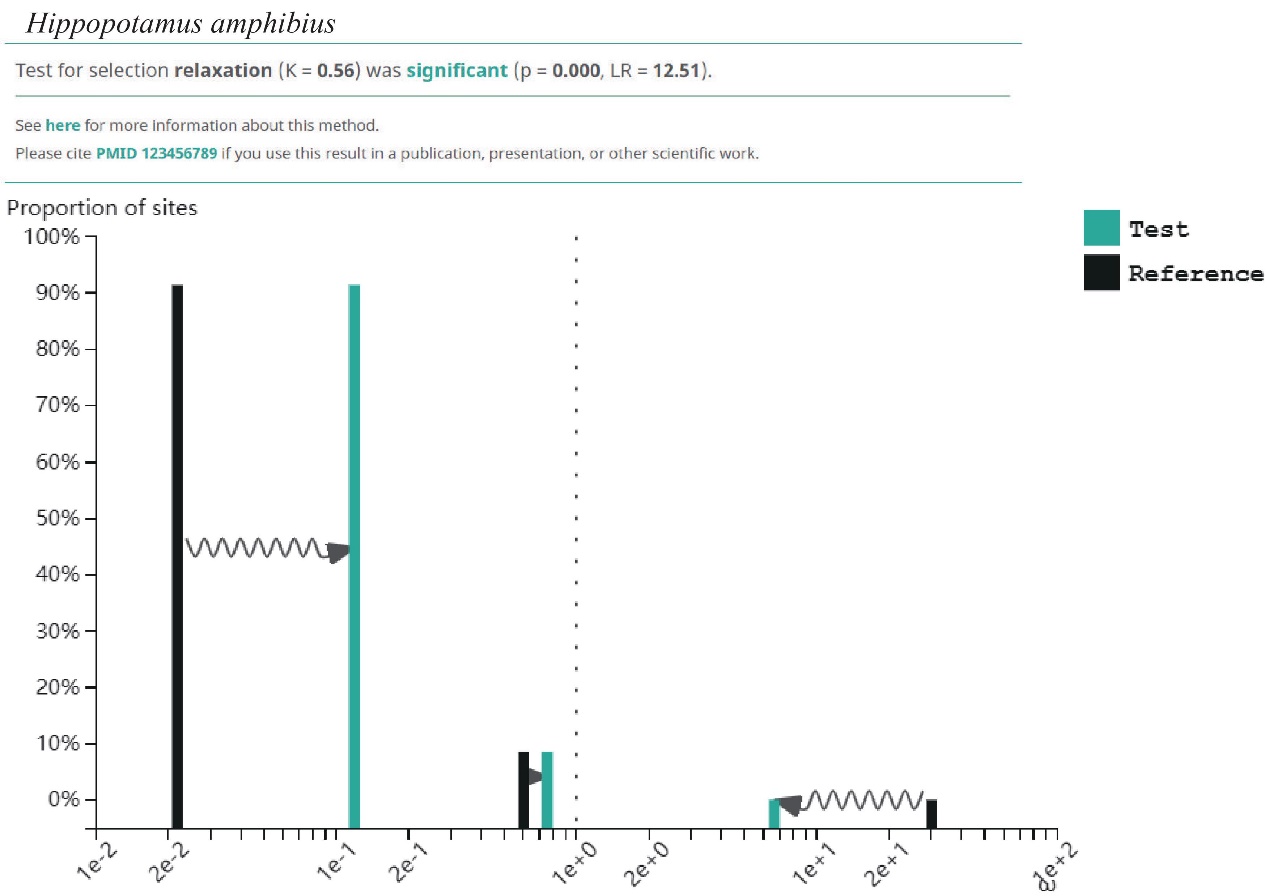

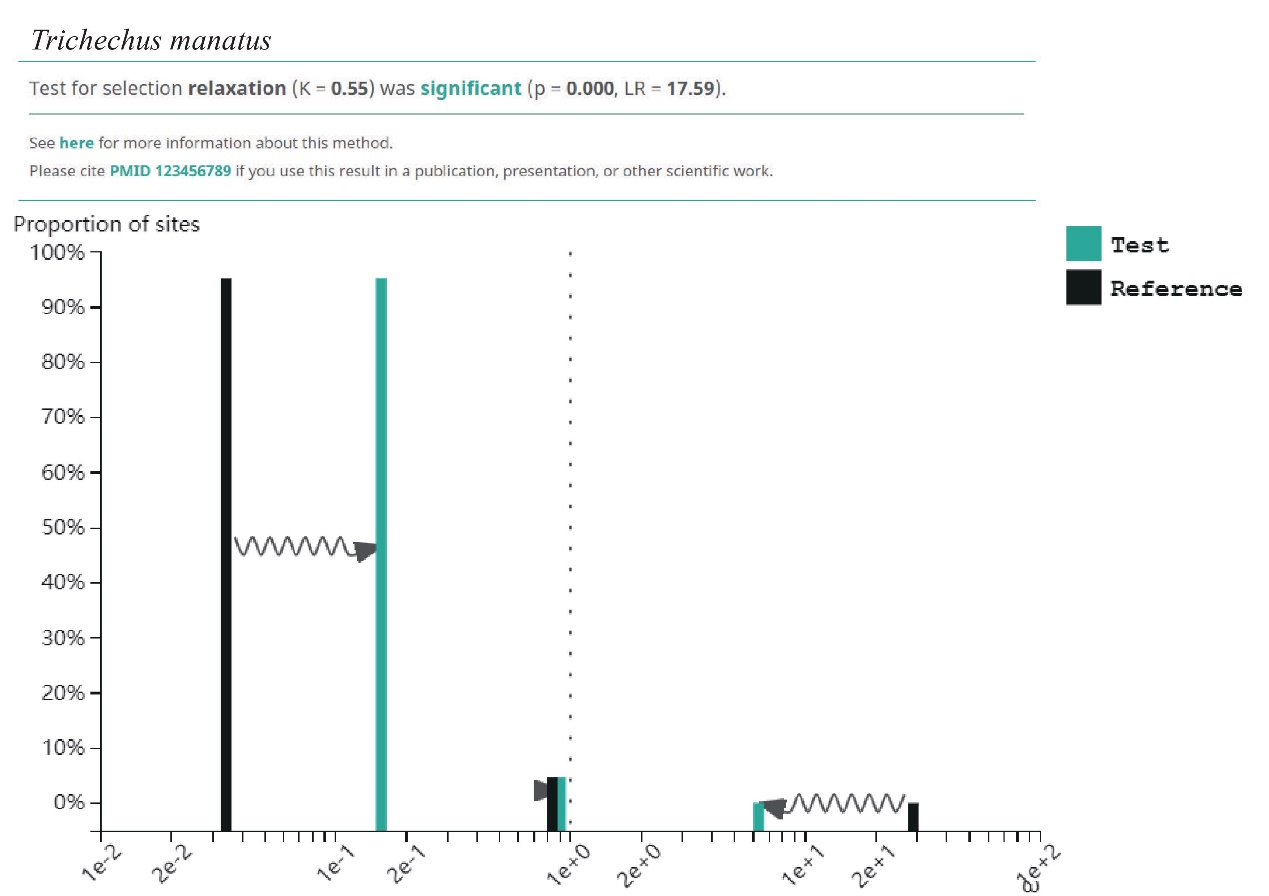

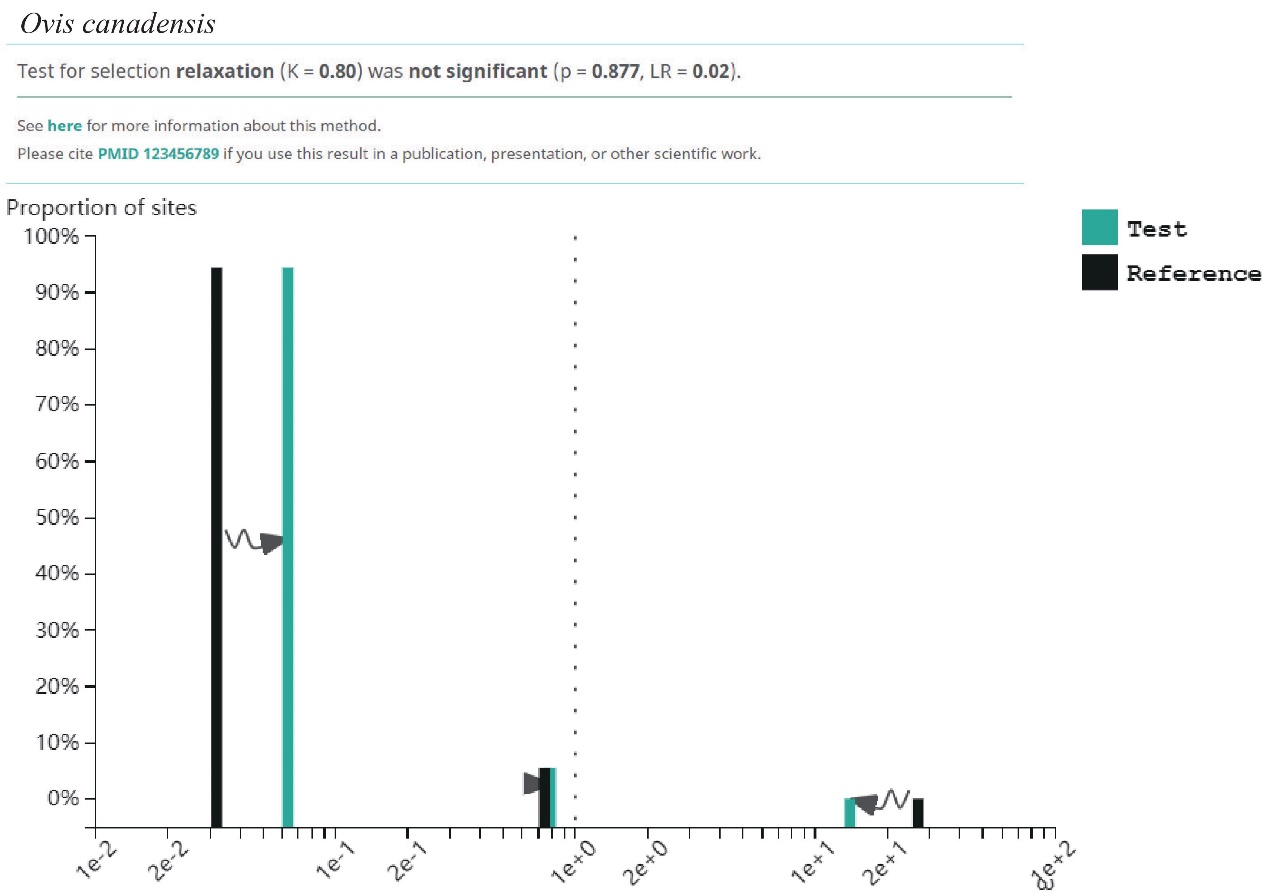


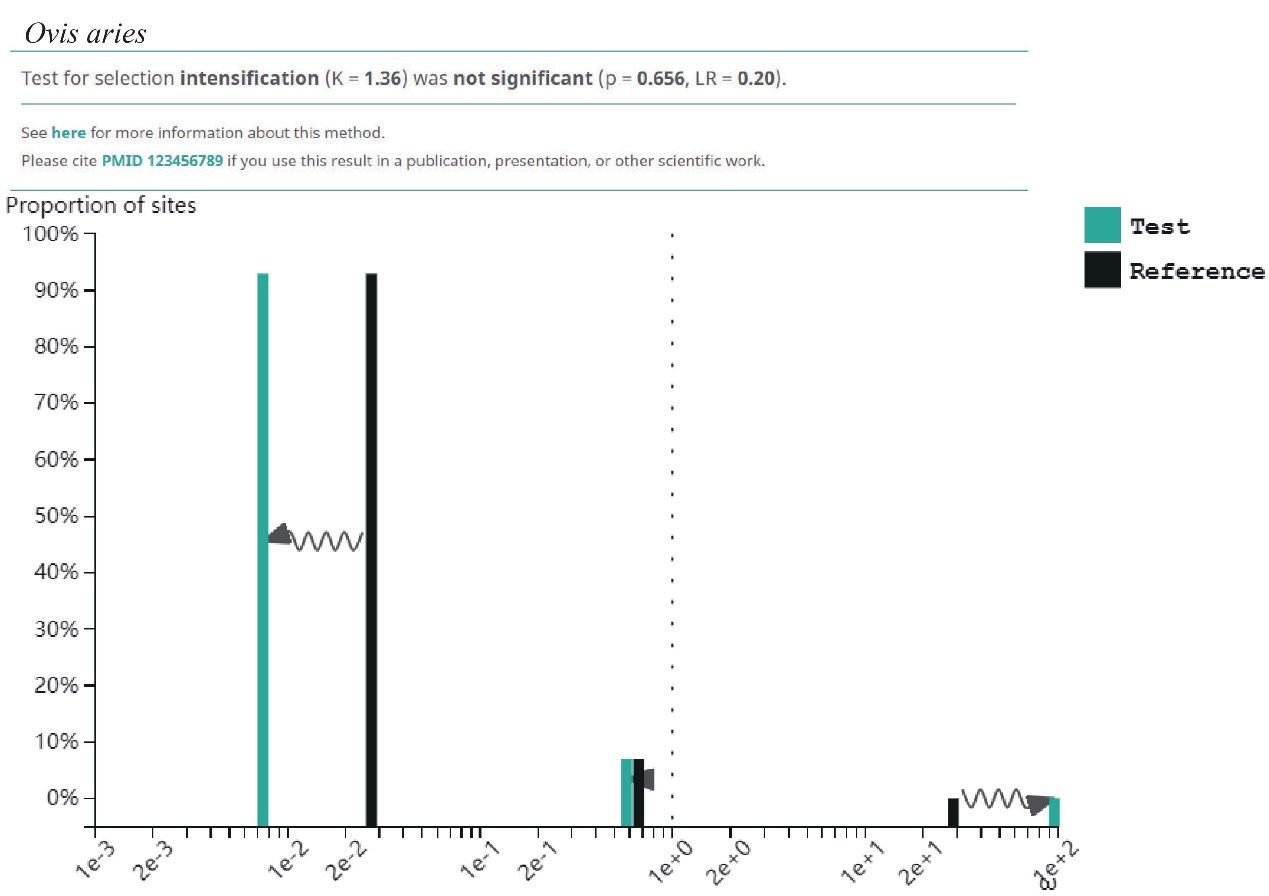


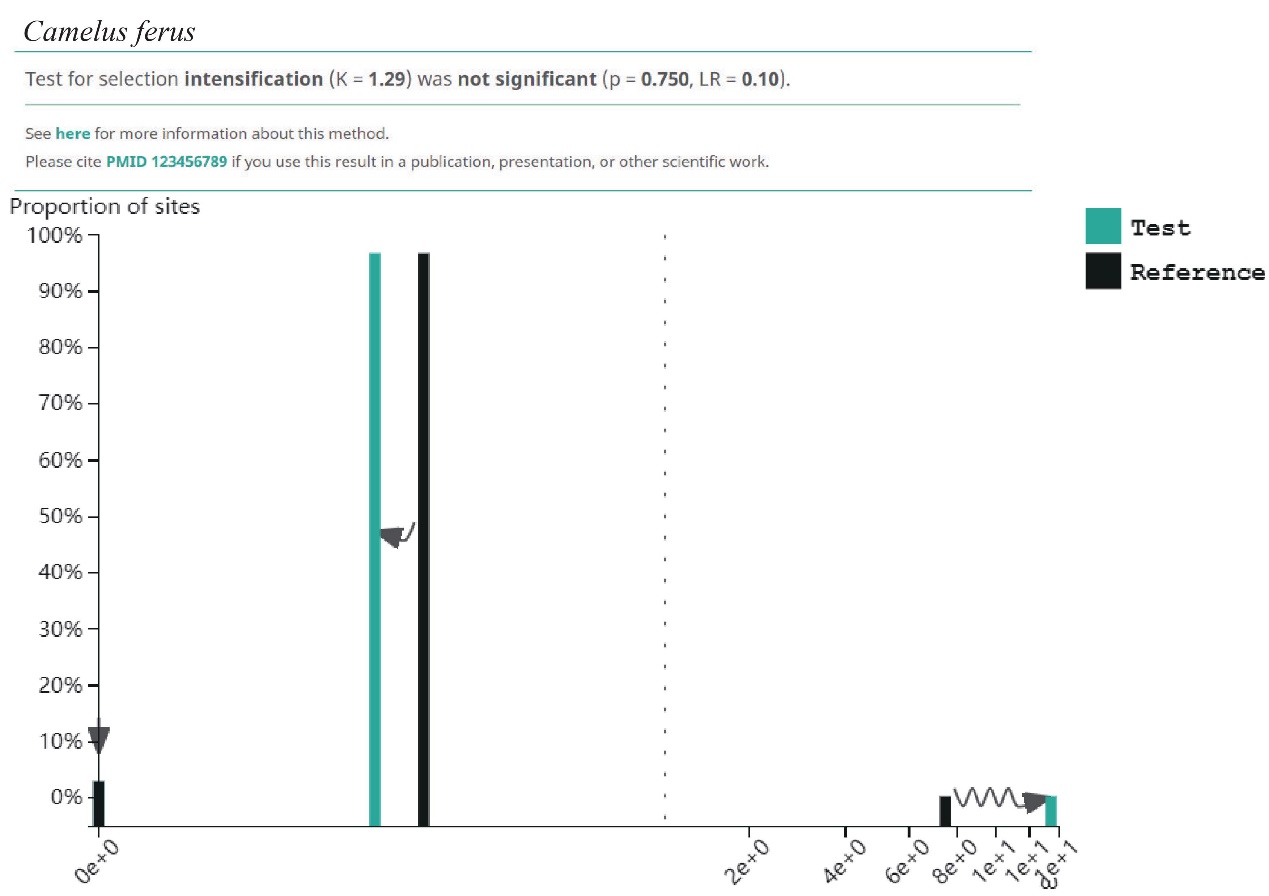


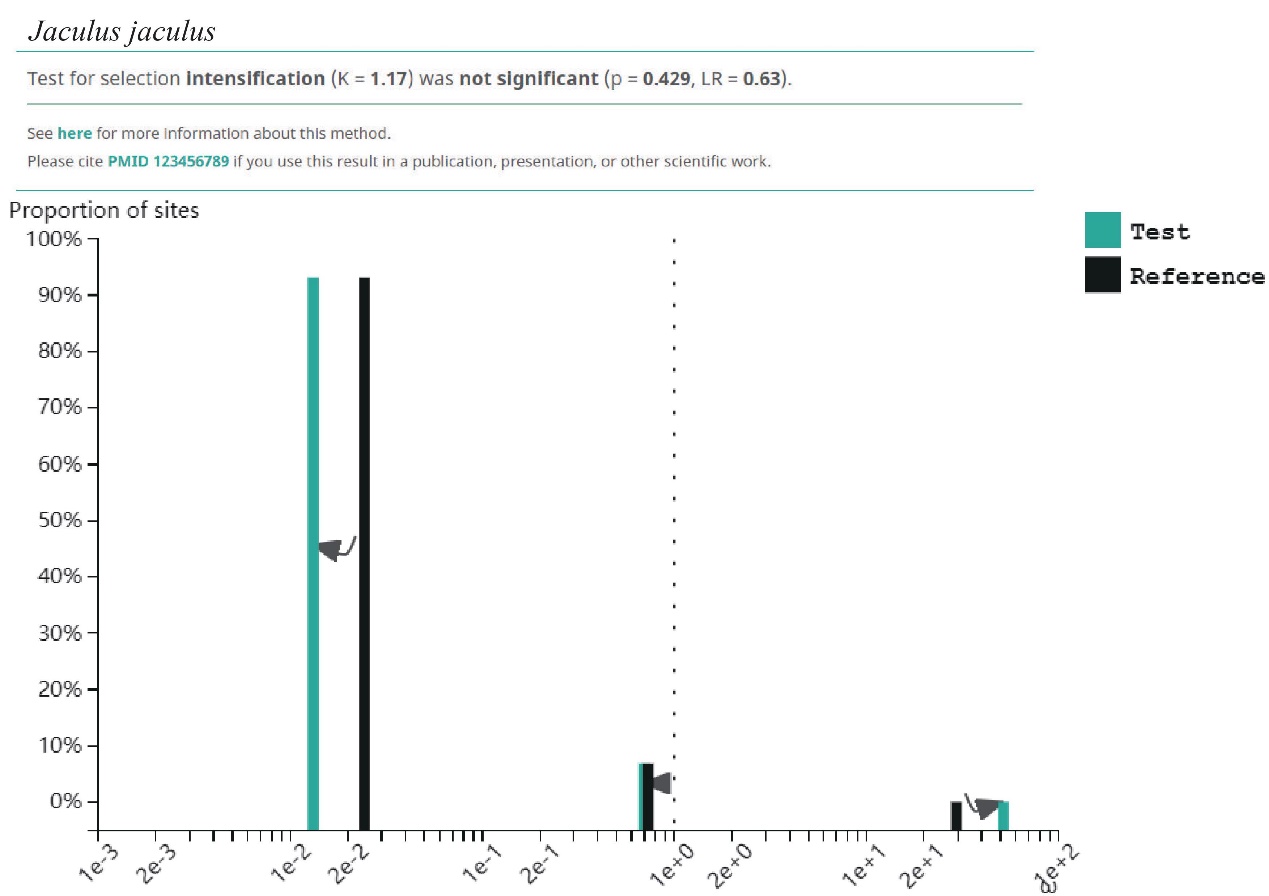


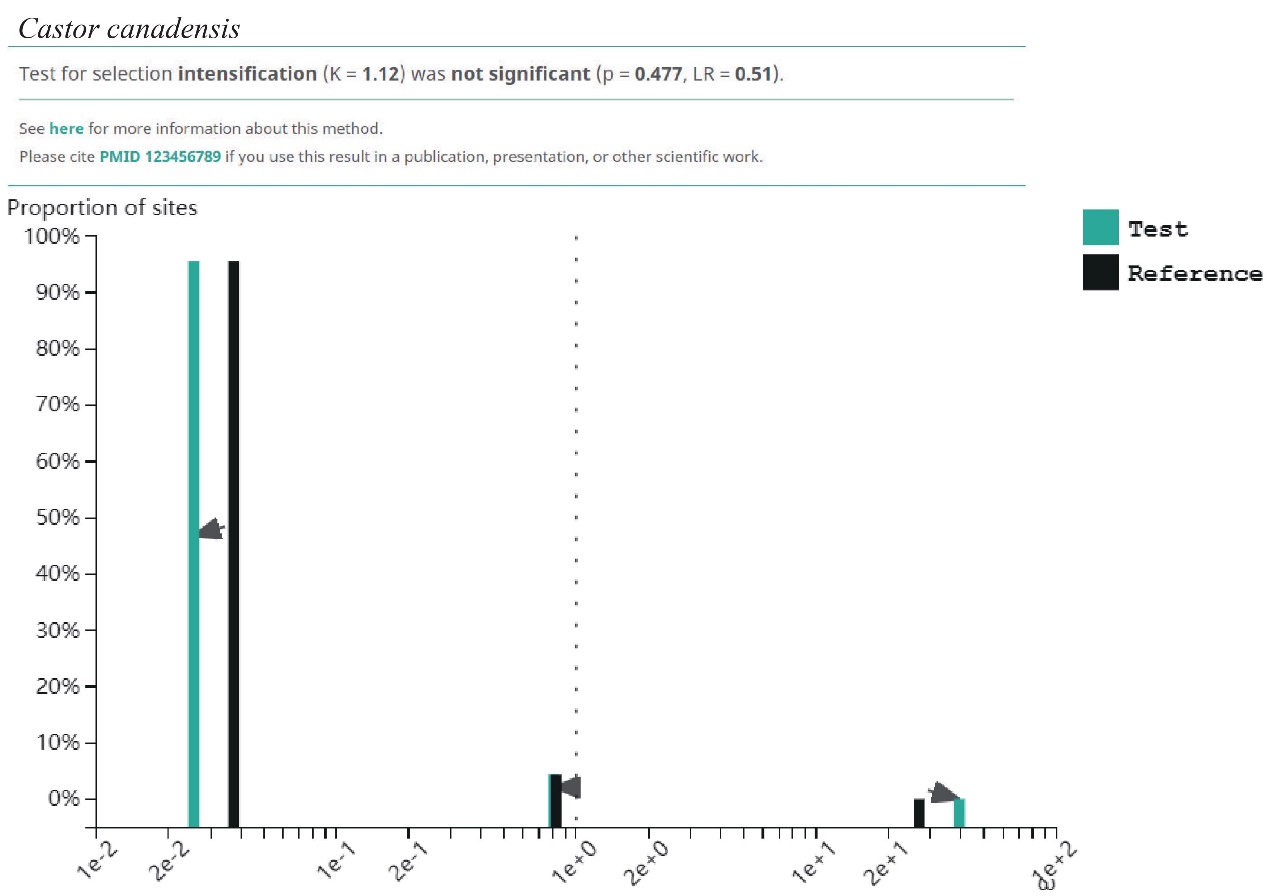


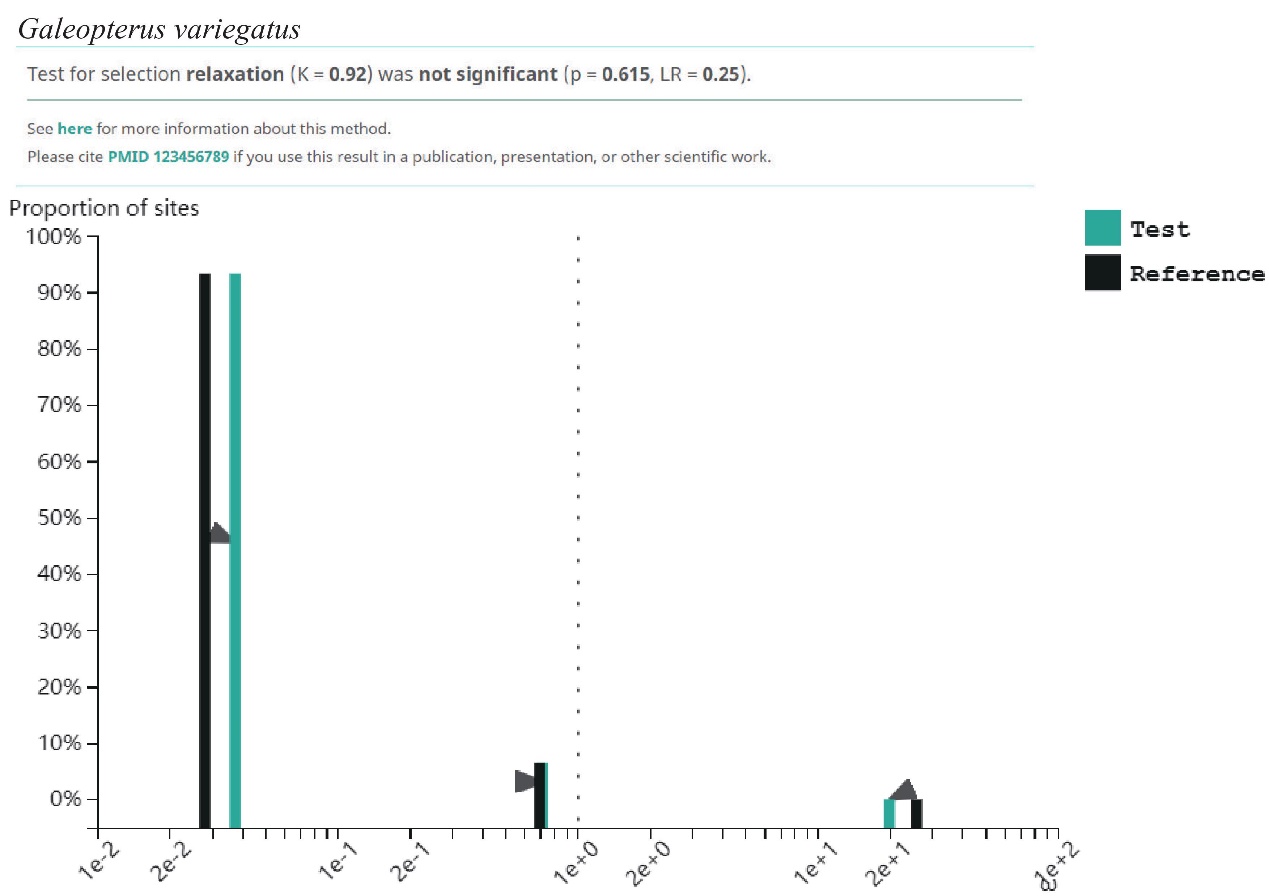

Supplement: Supplementary file 1 — Appendix S1 [file ECE3-12-e8731-s001.docx]
